# Compensatory effects of pointing and predictive cueing on age-related declines in visuospatial working memory

**DOI:** 10.3758/s13421-016-0611-1

**Published:** 2016-04-28

**Authors:** Kim Ouwehand, Tamara van Gog, Fred Paas

**Affiliations:** 1 Department of Psychology, Education & Child Studies, Erasmus University Rotterdam, P.O. Box 1738, 3000 DR Rotterdam, The Netherlands; 2 Department of Education, Utrecht University, Utrecht, The Netherlands; 3 Early Start Research Institute, University of Wollongong, Wollongong, New South Wales Australia

**Keywords:** Aging, Working memory, Gestures

## Abstract

In this study, we investigated whether the visuospatial working memory performance of young and older adults would improve if they used a multimodal as compared with a unimodal encoding strategy, and whether or not visual cues would add to this effect. In Experiment 1, participants were presented with trials consisting of an array of squares and an array of circles. They were instructed to point at one type of figure (multimodal encoding strategy) and only to observe the other (unimodal encoding strategy). After each trial, an immediate location recognition test of one of the two arrays followed. In Experiment 2, the same task was used, but a cue was provided, either before or after the encoding phase, indicating which of the two arrays would be tested. Our results showed that a multimodal, as compared with a unimodal, encoding strategy improved visuospatial working memory performance in both young and older adults (Exp. 1), and that adding visual cues to the multimodal but not to the unimodal encoding strategy improved older adults’ performance up to the level of young adults (Exp. 2). In both age groups, cueing *after* encoding led to higher performance in the multimodal than in the unimodal condition when the *second* array was tested. However, cueing *before* encoding led to higher performance in the multimodal than in the unimodal condition when the *first* array of the figure sequence was tested. These results suggest that pointing together with predictive cueing can have beneficial effects on visuospatial working memory, which is especially important for older adults.

## Introduction

In the present study, we investigated the effects of a multimodal (visual–motoric) versus a unimodal (visual-only) encoding strategy on visuospatial working memory in young and older adults. Because healthy aging has been associated with declines in working memory functioning (e.g., Salthouse & Babcock, 1991), it is important to find compensation strategies for older adults. Research on the enactment effect has already convincingly shown that a multimodal encoding strategy involving the motoric modality (enacting an action phrase) in addition to the auditory (listening to an action phrase) or visual (reading an action phrase) modality during encoding has positive effects on episodic memory (recall of action phrases) in young (e.g., Engelkamp, 1998; Nilsson, 2000; Zimmer, 2001; Zimmer & Engelkamp, 1999) and in older (Erngrund, Mäntylä, & Rönnlund, 1996; Feyereisen, 2009) adults. From a cognitive aging perspective this is interesting, because episodic memory declines with aging (Bastin & Van der Linden, 2005; Bayer et al., 2011; Spencer & Raz, 1995; Swick, Senkfor, & Van Petten, 2006; Trott, Friedman, Ritter, Fabiani, & Snodgrass, 1999). A possible explanation of the enactment effect is that an action encodes and stores the elements (the object and the action) in the sentence as an integrated event in memory (Kormi-Nouri & Nilsson, 2001).

Interestingly, Chum, Bekkering, Dodd, and Pratt (2007) showed that visuospatial working memory performance for figure locations that were manually pointed at during encoding was better than that for figure locations that were only visually observed. Participants were presented with a sequence of simple figures consisting of an array of squares and an array of circles, varying from three to five figures per array. Each figure was presented sequentially and disappeared after a fixed presentation time; the order of presentation of the two arrays was counterbalanced and randomized between trials. Participants were instructed to point at one type of figure (e.g., the squares; which type was also counterbalanced between participants) and only to visually observe the other type of figure. Immediately after presentation of the two arrays, a test phase followed in which a configuration of either squares or circles was shown, and participants had to judge whether or not the locations of the figures corresponded to the ones presented in the preceding sequence. Therefore, the time lag between encoding and the test phase varied depending on the order of array presentation during encoding. Chum et al. found that a multimodal (visual–motoric) encoding strategy led to better visuospatial working memory performance than did a unimodal (visual-only) encoding strategy, and participants performed better on test trials regarding the second array of figures than for the array presented first. Moreover, they found an interaction between encoding strategy and array size. Specifically, the interaction effect showed that the beneficial effect of a multimodal encoding strategy declined with increasing array size, and even disappeared for the largest arrays (five squares and five circles).

One of the explanations for the facilitating effect of pointing on visuospatial working memory provided by Chum et al. (2007) was based on the selection-for-action hypothesis (Allport, 1989). This hypothesis holds that stimuli that we intend to act upon receive more attention than stimuli that we do not intend to act upon. Hence, stimuli that require an action would be processed and encoded better. Furthermore, more recent evidence suggests that this attentional bias is related to whether or not the stimuli are perceived near the hands. If stimuli are perceived near the hands, beneficial effects on performance are found on all kinds of tasks involving cognitive control processes, such as spatial attention (Reed, Grubb, & Steele, 2006), visual working memory (Tseng & Bridgeman, 2011), and executive functioning (Weidler & Abrams, 2014). Note that with pointing, the hand is brought in close proximity to the stimuli, and the evidence described above showed that this enhances performance on all kinds of cognitive tasks, including visual working memory. However, the above-discussed studies all used young adults as participants, and to the best of our knowledge, no studies have been conducted with older adults yet. We suggest that these findings are especially relevant for older adults because perceptual ability (Schneider & Pichora-Fuller, 2000), focused attention (Rösler, Mapstone, Hays-Wicklund, Gitelman, & Weintraub, 2005), working memory (Salthouse & Babcock, 1991), and executive functioning (Salthouse, Atkinson, Berish, & Diane, 2003) decline with aging.

The finding that the positive effect of pointing was smaller when the arrays tested were larger is possibly related to the limited capacity of working memory. Working memory is known to have a capacity of around three to five items when processing information (Cowan, 2010). It would make sense that the effect of encoding strategy would disappear if the number of items to be remembered exceeded this limited working memory capacity (i.e., cognitive overload; see Paas, Tuovinen, Tabbers, & Van Gerven, 2003). As for the effect that participants performed better when the second than when the first array was tested (effect of order), Chum et al. (2007) called this “a typical effect of temporal proximity,” meaning that memory was improved because the time lag between the encoding and test phase of the second array was shorter than that between the encoding and test phase of the first array. However, in the present study we propose an alternative explanation, that in trials in which the first array was tested, performance not only suffered from the time lag, but also from retroactive interference. *Retroactive interference* occurs when the learning of new information interferes with the memory for older information (e.g., Ebert & Anderson, 2009). That means that in the paradigm of Chum et al., memory for the first array was disturbed by the encoding of the second array.

Inhibitory functions of working memory are important in order to deal adequately with interference (e.g., irrelevant information). We found that older adults have more problems with inhibiting irrelevant information than do young adults (e.g., Houx, Jolles, & Vreeling, 1993; Stoltzfus, Hasher, Zacks, Ulivi, & Goldstein, 1993). However, this effect of age might depend on which type of inhibition is required. According to Hasher, Zacks, and May (1999), working memory has three inhibitory functions—namely, *access*, *deletion* (or *suppression*), and *restraint*. Relevant for the present study are the access and deletion functions. The *access* function involves the inhibition of irrelevant stimuli from entering working memory, and the *deletion* function involves the selective deletion of irrelevant stimuli after these have entered working memory.

In their study, Cansino, Guzzon, Martinelli, Barollo, and Casco (2011) investigated these inhibitory functions in young and older adults with a visuospatial working memory task. In this task, participants saw a sequence of two circles consisting of Gabor elements in which one or more of the Gabor elements were missing. In the test phase, participants were presented with a circle similar to the one in the encoding phase. Participants had to judge whether or not the test circle was missing the same Gabor element(s) as one of the circles presented in the preceding encoding phase. In the test conditions, participants received cues presented either before (“access” condition) or after (“deletion” condition) the encoding phase, indicating which of the two circles was task-relevant. In the control conditions, blank cues that did not provide information on the task relevance were presented either before or after the encoding phase. When Cansino et al. compared the test conditions with the control conditions, cueing relevance improved young adults’ performance in both the access and the deletion conditions, but the performance of older adults only improved when relevance was cued *before* encoding. These results suggest that older adults have no trouble filtering out or ignoring irrelevant information before it can access working memory, but have problems suppressing (i.e., deleting) this information after it has accessed working memory (Cansino et al., 2011).

We suggest that, because in Chum et al.’s (2007) study the task relevance became clear after stimulus presentation, this paradigm required only the deletion function. This means that the irrelevant array needed to be suppressed after it had entered working memory. Therefore, it seems that the effect of order might be influenced not only by the time lag, but also by retroactive interference (i.e., the presentation of the second, irrelevant array). Although the arrays consisted of three figures, participants had to keep six figures in working memory until the test phase. Because Chum et al. found that the effect of pointing was larger under conditions of lower working memory load, and Cansino et al. (2011) showed that cueing relevance can enhance visuospatial working memory performance in both young and older adults, we suggest that cueing relevance can allow participants to offload working memory, and thereby improve working memory performance. Because older adults have suboptimal working memory functioning when compared with young adults (Salthouse & Babcock, 1991), holding six items in working memory (as in the simplest condition in Chum et al.’s study) might be more challenging for this population. Therefore, we suggest that cueing (especially cueing before encoding; see Cansino et al., 2011) could increase the effect of pointing in young, but especially in older, adults.

### The present study

The present study consisted of two experiments. In Experiment 1, the paradigm of Chum et al. (2007) was used to explore whether a multimodal (visual–motoric) encoding strategy would lead to better visuospatial working memory performance than a unimodal (visual-only) strategy—not only for young, but also for older, adults. Because aging is also related to reduced cognitive speed (Salthouse, 1996), which is reflected in lower perceptual and psychomotor speed (Salthouse, 2000), it is important to mention that the pointing condition of the present task required both of these processes. The participants in Chum et al.’s study needed to detect each figure that randomly appeared at different locations (relying on perceptual speed), and then to point to the figure within 1,000 ms (relying on psychomotor speed). Because we were not sure whether older adults could keep up with the 1,000-ms display time (in which case, the performance of the older adults would suffer), trials with a display time of 1,500 ms per figure were added in Experiment 1. Adding this Display Time factor was purely explorative, and we do acknowledge that this longer display time might also have negative effects on performance, because of the temporal limitations of working memory. We hypothesized that the effects of encoding strategy and order found by Chum et al. would be replicated in young adults, and that these findings would also extend to older adults. In addition, because Chum et al. found that the effect of encoding strategy was most pronounced for the smallest arrays (three figures per array) and the effect of encoding was smaller for the larger arrays, we only used trials with three figures per array in Experiment 1.

In Experiment 2, visual cues presented either before (targeting the access function) or after (targeting the deletion function) the encoding phase were added to the task used in Experiment 1. First, because Chum et al. (2007) found that the positive effect of a multimodal encoding strategy (i.e., visual–motoric) was most pronounced in the condition with the lowest working memory load (three figures per array), and Cansino et al. (2011) found that cueing relevance can offload visuospatial working memory, we hypothesized that cueing would positively moderate the effect of multimodal encoding on visuospatial working memory performance. In addition, given the age-related decline in working memory, the older adults were expected to benefit more from multimodal encoding than would the young adults. Second, we hypothesized that young and older adults’ performance would be improved when a cue was provided before the encoding phase (targeting the access condition), but only young adults’ performance would be improved by a cue provided after encoding (targeting the deletion function; see Cansino et al., 2011). Although we do acknowledge the explanation for the effect of order given by Chum et al. (difference in the time lag between encoding and test), we suggest that retroactive interference (i.e., the presentation of new information interfering with memory for older information) also might have influenced the effect of order. Therefore, we hypothesized that cueing relevance, especially before encoding (because then the second array, which was the “new information,” could be ignored before it entered working memory), would enhance memory on trials with the highest retroactive interference (when the first array was test relevant), and thereby reduce the effect of order.

## General method

### Materials and procedure

All tasks were programmed in E-Prime 2.0 and presented on a 17-in. ELO touchscreen with a 1,024 × 768 resolution, tilted backward at an angle of 30**°**.

#### Experimental task

Participants were tested in individual sessions, and the task took about 15 min to complete. The task started with a short training phase, in which participants were familiarized with the procedure of the trials. The encoding phase of each trial showed a figure sequence consisting of two arrays of three figures—that is, three white-filled circles and three white-filled squares. Half of the participants were instructed to point at the squares and only to look at the circles, and the other half were instructed to point at the circles and only to look at the squares. The presentation order (i.e., circles or squares first) was counterbalanced. The figures (i.e., square or circle) in each array were presented sequentially in one of 20 possible positions on the screen, and each location was used only once, in the encoding phase of a single trial. The figures disappeared when they had been pointed at, or after a maximum display time of either 1,000 ms (as in Chum et al., 2007) or 1,500 ms. After the presentation of the two arrays in the encoding phase of a trial, a mask was presented for 150 ms. Next, the test phase followed, showing a configuration of three white-filled circles or squares. Participants had to judge whether or not the figures were positioned at the locations at which they had been presented in the encoding phase, by pressing the word “correct” (i.e., as seen in the encoding phase) or “incorrect” on the touchscreen.

**Fig. 1:**
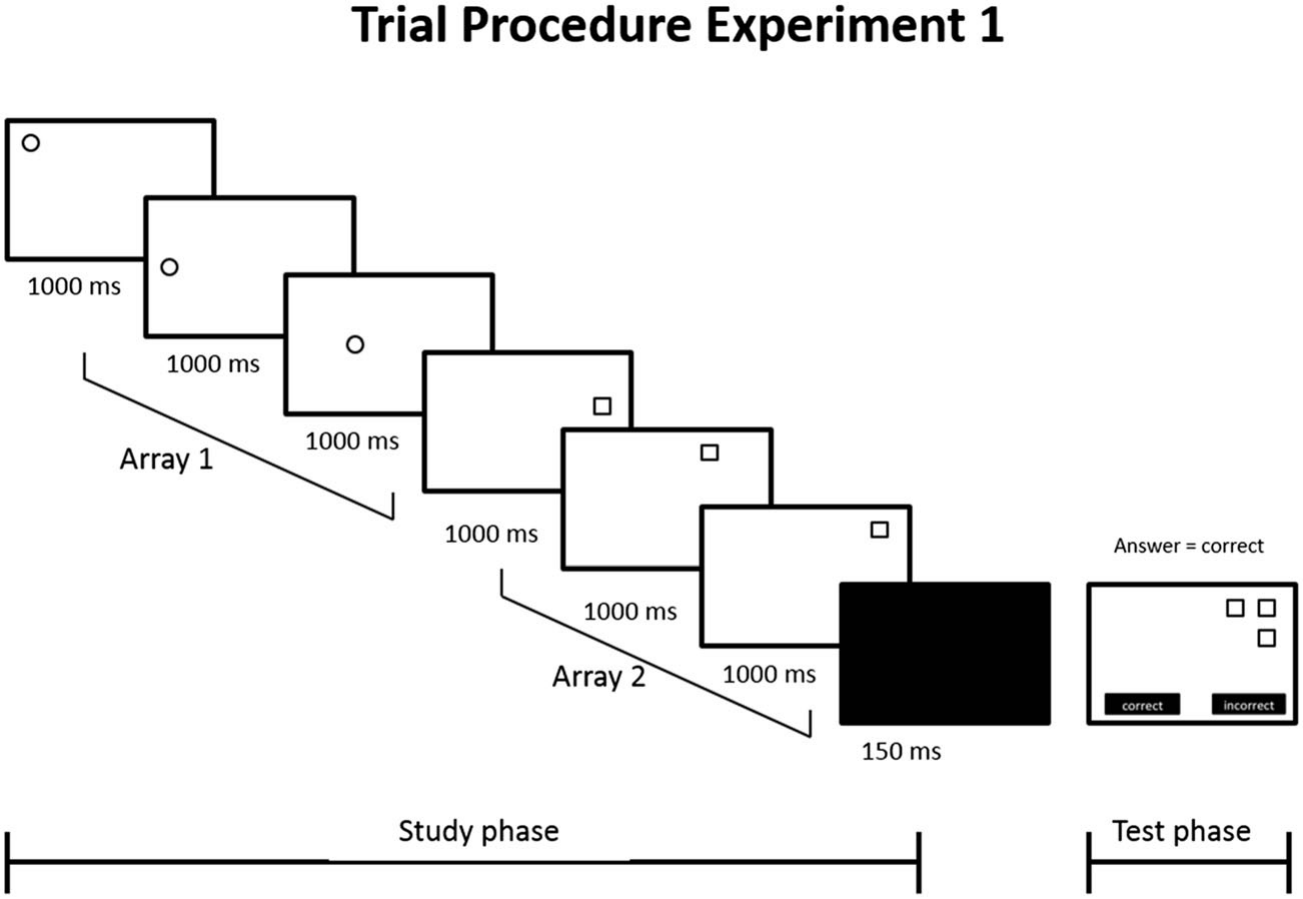
Trial procedure of Experiment 1 in a trial with a 1,000-ms display time. Trials presented with the 1,500-ms display time condition were the same, except that all items were displayed for 1,500 ms

The total test consisted of 64 trials; in 32 trials, the test-relevant array was encoded multimodally, by both looking and pointing (visual–motoric), and in the other 32 trials, the test-relevant array was encoded unimodally (visual only). In half of the 32 trials per encoding strategy, the first array was test-relevant, in the other half, the second array. Of the 32 trials per encoding strategy, in 16 trials each figure was presented for 1,000 ms, and in 16 each figure was presented for 1,500 ms. Overall, 50 % of the test trials had to be answered with “correct,” and 50 % with “incorrect.” After this response, the next trial started. Figure 1 depicts the trial procedure of a trial with a figure display time of 1,000 ms.

### Data analysis

For all omnibus analyses, a significance level of .05 was used. On the follow-up analyses of the omnibus tests, a Bonferroni correction was applied. This means that the significance level of .05 was divided by the total number of follow-up analyses for each experiment (i.e., in Exp. 1 the corrected significance level for the results of the follow-up tests on accuracy was .05/6 = .008; in Exp. 2, the corrected significance levels were .05/8 = .006 for accuracy, and .05/4 = .013 for reaction times). Partial eta-squared (*η*_p_^2^) was calculated as a measure of effect size, with values of .01, .06, and .14, respectively, being considered to characterize small, medium, and large effect sizes (Cohen, 1988). Because we expected that older adults might be slower in their pointing response in the encoding phase, and because we did not know whether they could keep up with the 1,000-ms stimulus display time in the pointing condition, the reaction times for pointing in the encoding phase were compared between the young and older adults in Experiment 1. Test performance in both the multimodal and unimodal encoding conditions was determined by accuracy, expressed as the percentage of accurate judgments in the test phase (i.e., pressing “correct” when the configuration shown in the test phase was the same as during encoding, or pressing “incorrect” when it was not), and by mean reaction times, in milliseconds. Participants who had an average performance below chance level (<50 %) or an average reaction time higher than 3,000 ms were excluded from the analyses.

## Experiment 1

### Method

#### Participants

Here the participants were 39 young adults (28 women, 11 men; *M*_age_ = 20.8 years, *SD* = 2.1, age range 18–26 years), who were all students enrolled at a Dutch university, and 38 older adults (23 women, 15 men; *M*_age_ = 67.1 years, *SD* = 4.3, age range 60–83 years), who had been recruited via advertisements in community centers and local newspapers. The advertisements called for healthy older adults (>60 years of age), and during admission, participants were asked whether they had experienced a stroke (CVA or TIA), dementia, other cognitive problems, or any kind of brain damage or (mild) head trauma in the past. Participants who answered “yes” to one of these questions were not included in the sample. The young adults received course credit, and the older adults received a small monetary reward (€7.50) for their participation.

#### Materials and procedure

Prior to the experimental task described in the General method section, a computerized operation span task (Unsworth, Heitz, Schrock, & Engle, 2005) was administered in order to obtain a general measure of the cognitive functioning of both age groups. These types of working memory span tasks have been found to predict performance on a wide range of cognitive tasks (Engle, Tuholski, Laughlin, & Conway, 1999; Kane, Bleckley, Conway, & Engle, 2001) and share a large amount of variance, indicating that they measure the same construct (Unsworth et al., 2005). Although a large body of evidence has indicated that older adults show age-related cognitive decline relative to young adults (Cabeza & Dennis, 2013; Conway et al., 2005), this measure was taken to check whether this was also the case in the present sample.

In this task, participants were presented with arrays of letters intermixed with arithmetic problems they had to solve. Each trial started with a letter, followed by a problem, followed by a letter, and so forth. In total, 75 letters and 75 problems were presented in trials varying randomly in length from three to seven letter–problem pairs. One point was assigned for each letter that was recalled in the correct position in the array, which could result in a maximum score of 75.

### Results

#### Operation span task

An analysis of variance (ANOVA) showed a significant difference in operation span scores between young and older adults, *F*(1, 75) = 26.72, *MSE* = 265.52, *p* < .001, *η*_p_^2^ = .26, with, as expected, operation span being higher in young adults (*M* = 41.41, *SD* = 18.54) than in older adults (*M* = 22.21, *SD* = 13.61). The operation span score showed no significant correlation with the mean performance accuracy on the experimental task of the young (*r* = .255, *p* = .117) or the older (*r* = .237, *p* = .152) adults.

#### Experimental task

##### Encoding

Older participants (*M* = 668.14 ms, *SD* = 54.67) were slower to point to the figures than were the young adults (*M* = 603.61 ms, *SD* = 54.68), *F*(1, 75) = 26.81, *MSE* = 2,989.14, *p* < .001, *η*_p_^2^ = .26.

##### Test

Accuracy and reaction time data were analyzed by means of a mixed 2 × 2 × 2 × 2 repeated measures ANOVA with the within-subjects factors Encoding Strategy (multimodal vs. unimodal), Order (first vs. second array relevant), and Presentation Time (1,000 vs. 1,500 ms), and the between-subjects factor Age Group (young vs. older adults). All means and standard deviations for accuracy (as percentages) and reaction times (in milliseconds) of Experiment 1 can be found in Table 1.

**Table 1:** Means (and *SD*s) for accuracy (Acc) and reaction times (RT) in Experiment 1

			Young Adults (*n* = 39)				Older Adults (*n* = 38)			
			1,000 ms		1,500 ms		1,000 ms		1,500 ms	
	Order		*M*	*SD*	*M*	*SD*	*M*	*SD*	*M*	*SD*
Pointing	1st	Acc (%)	76.59	13.96	78.74	14.83	63.39	16.48	69.97	16.89
	1st	RT (ms)	1,606	433	1,795	512	2,049	813	2,077	535
	2nd	Acc (%)	91.59	8.07	91.15	13.63	83.18	13.40	80.84	13.45
	2nd	RT (ms)	1,515	458	1,606	433	1,681	404	1,695	443
Observing	1st	Acc (%)	73.67	16.78	75.82	15.89	63.74	12.15	60.16	16.23
	1st	RT (ms)	1,835	566	1,935	683	2,162	572	2,185	597
	2nd	Acc (%)	87.38	11.24	88.69	10.04	71.55	19.48	75.87	17.44
	2nd	RT (ms)	1,425	390	1,525	501	1,776	364	1,841	455

For reasons of readability and manuscript length, only significant effects are discussed here; statistics for all of the analyses in Experiment 1 can be found in Tables 2 and 3 (accuracy) and 4 (reaction times).

**Table 2:** Statistics of the analyses on performance accuracy in Experiment 1

Analysis	Factor(s)	*df*	*MSE*	*F*	*p*	*η*_p_^2^
Omnibus test	**A**	**1, 75**	**0.04**	**49.39**	**<.001**	**.44**
(E × O × T × A)	**E**	**1, 75**	**0.02**	**16.89**	**<.001**	**.18**
	E × A	1, 75		2.09	.153	.03
	**O**	**1, 75**	**0.02**	**188.43**	**<.001**	**.72**
	O × A	1, 75		<0.01	.981	<.01
	T	1, 75	0.03	0.98	.326	.01
	T × A	1, 75		<0.01	.984	<.01
	E × O	1, 75	0.02	0.93	.339	.01
	E × O × A	1, 75		0.58	.448	<.01
	E × T	1, 75	0.02	0.05	.827	<.01
	E × T × A	1, 75		0.43	.514	<.01
	O × T	1, 75	0.02	0.26	.609	<.01
	O × T × A	1, 75		0.08	.782	<.01
	**E × O × T**	**1, 75**	**0.02**	**5.14**	**.026**	**.06**
	E × O × T × A	1, 75		3.39	.070	.04
Follow-up 1.1: T = 1,000 ms	**A**	**1, 77**	**0.03**	**33.76**	**<.001**	**.31**
	**E**	**1, 77**	**0.02**	**9.10**	**<.001**	**.11**
	E × A	1, 77		0.46	.498	<.01
	**O**	**1, 77**	**0.02**	**93.41**	**<.001**	**.56**
	O × A	1, 77		0.04	.849	<.01
	E × O	1, 77	0.09	5.33	.024	.07
	E × O × A	1, 77		3.46	.067	.04
Follow-up 1.2: T = 1,500 ms	**A**	**1, 77**	**0.04**	**29.18**	**<.001**	**.28**
	**E**	**1, 77**	**0.20**	**10.49**	**<.001**	**.12**
	E × A	1, 77		2.28	.135	.03
	**O**	**1, 77**	**0.02**	**77.19**	**<.001**	**.51**
	O × A	1, 77		0.05	.827	<.01
	E × O	1, 77	0.02	0.81	.371	.01
	E × O × A	1, 77		0.55	.459	<.01
Follow-up 2.1: T = 1,000 ms; O = First	E	1, 78	0.02	0.30	.587	<.01
**Follow-up 2.2: T = 1,000 ms; O = Second**	**E**	**1, 78**	**0.02**	**14.59**	**<.001**	**.16**
**Follow-up 2.3: T = 1,500 ms; O = First**	**E**	1, 78		**8.95**	**.004**	**.10**
Follow-up 2.4: T = 1,500 ms; O = Second	E	1, 78	0.02	3.69	.058	.05

A = Age Group (young vs. older adults); E = Encoding Strategy (pointing vs. observation only); T = Display Time (1,000 vs. 1,500 ms); O = Order (test stimulus is first vs. second array). Significant effects are printed in boldface

**Table 3:** Statistics of the analysis on performance accuracy for only the young adults in Experiment 1

Analysis	Factor(s)	*df*	*MSE*	*F*	*p*	*η*_p_^2^
Young adults	**E**	**1, 38**	**0.02**	**4.99**	**.032**	**.12**
(E × T × O)	T	1, 38	0.02	0.70	.408	.02
	**O**	**1, 38**	**0.02**	**95.05**	**<.001**	**.71**
	E × T	1, 38	0.02	0.10	.751	<.01
	E × O	1, 38	0.02	0.02	.889	<.01
	T × O	1, 38	0.01	0.42	.523	.01
	E × T × O	1, 38	0.01	0.11	.744	<.01

E = Encoding Strategy (pointing vs. observation only); T = Display Time (1,000 vs. 1,500 ms); O = Order (test stimulus is first vs. second array). Significant effects are printed in boldface

**Table 4:** Statistics of the analysis on reaction times in Experiment 1

Analysis	Factor(s)	*df*	*MSE*	*F*	*p*	*η*_p_^2^
Omnibus test	**A**	**1, 75**	**1,129,431.82**	**12.31**	**.001**	**.14**
(E × T × O × A)	**E**	**1, 75**	**176,084.96**	**9.67**	**.003**	**.11**
	E × A	1, 75		0.10	.765	.01
	**T**	**1, 75**	**187,325.91**	**4.75**	**.032**	**.06**
	T × A	1, 75		1.55	.216	.02
	**O**	**1, 75**	**237,036.63**	**77.33**	**<.001**	**.51**
	O × A	1, 75		0.41	.522	<.01
	E × T	1, 75	102,919.18	0.02	.881	<.01
	E × T × A	1, 75		0.37	.547	<.01
	E × O	1, 75	159,377.15	1.74	.191	.02
	E × O × A	1, 75		2.14	.147	.03
	T × O	1, 75	96,136.12	0.13	.722	<.01
	T × O × A	1, 75		0.40	.529	<.01
	E × T × O	1, 75	88,014.38	0.67	.416	<.01
	E × T × O × A	1, 75		0.05	.824	<.01

A = Age Group (young vs. older adults); E = Encoding Strategy (pointing vs. observation only); T = Display Time (1,000 vs. 1,500 ms); O = Order (test stimulus is first vs. second array). Significant effects are printed in boldface

The analysis of the performance accuracy data showed main effects of encoding strategy (multimodal > unimodal), order (2nd array test-relevant > 1st array test-relevant), and age group (young > older), but not of presentation time. However, the main effects of encoding strategy and order were qualified by a three-way interaction between time, encoding strategy, and order. No other interaction effects were found (see Table 2, Omnibus test).

**Fig. 2:**
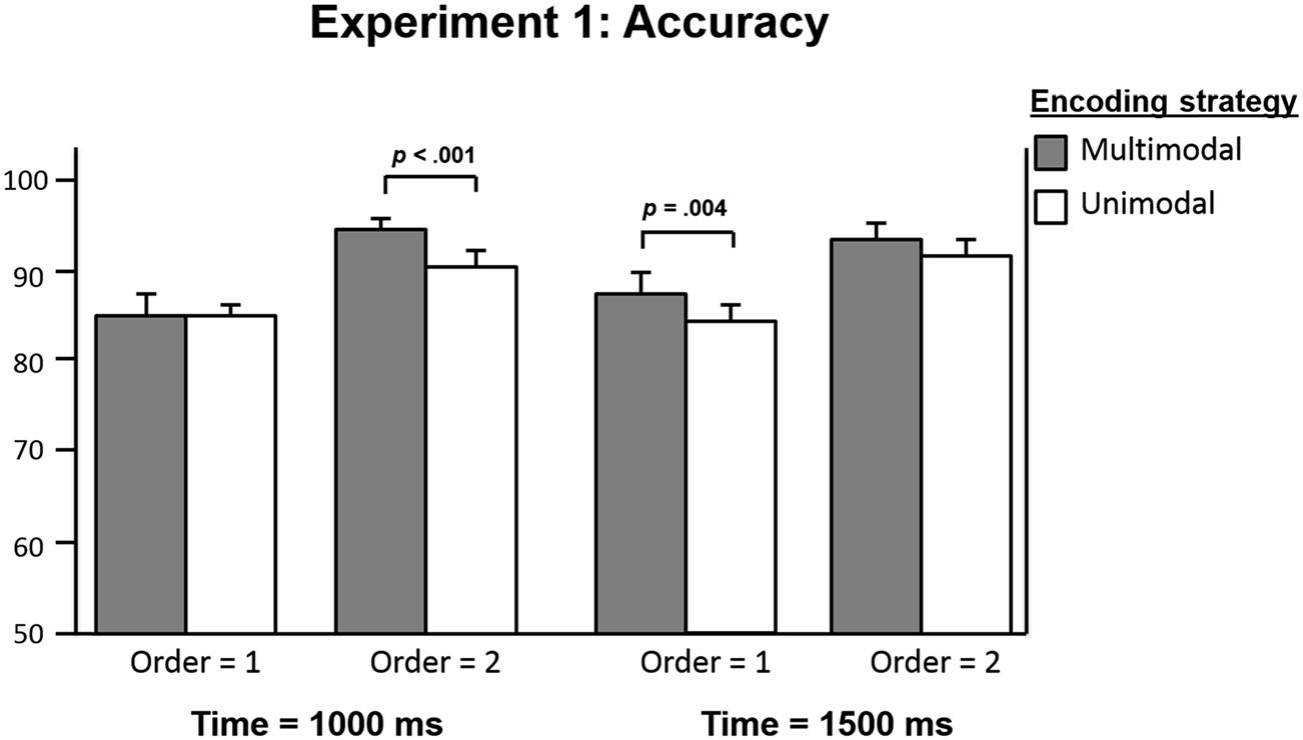
Experiment 1: Interaction between encoding strategy, time, and order

Because Presentation Time was not a factor in the original paradigm of Chum et al. (2007), this interaction was followed up on by analyzing performance separately on trials with the 1,000-ms display time per figure (as in the original paradigm) and the 1,500-ms display time per figure, with 2 (Encoding Strategy) × 2 (Order) ANOVAs. In line with the findings of Chum et al., the first analysis (time = 1,000 ms) yielded main effects of encoding strategy and order, but no interaction (see Table 2, Follow-up 1.1). The second analysis (time = 1,500 ms) also yielded a main effect of encoding strategy and order, but no interaction effects (see Table 2, Follow-up 1.2). Thus, the interaction between time, encoding strategy, and order could not be explained by the different display times. From visual inspection of the data, the effects of encoding strategy seemed to differ between the display time and order conditions (see Fig. 2). Therefore, four ANOVAs were conducted for the effect of encoding strategy: one for each combination of display time and order. The results showed significant effects of encoding strategy for performance on trials with the 1,000-ms display time in which the second array was test-relevant, and on trials with the 1,500-ms display time in which the first array was test-relevant. No effect of encoding strategy was found for performance on trials with the 1,000-ms display time in which the first array was test-relevant or on trials with the 1,500-ms stimulus display time in which the second array was test-relevant (see Table 2, Follow-ups 2.1–2.4).

Although we found no interactions with age group, we felt it would be relevant to conduct an exploratory follow-up analysis for only the young adults’ performance on trials with the 1,000-ms display time, to find out whether or not we would replicate the findings of Chum et al. (2007). As in the study by Chum et al., our analysis of the young adults’ performance showed main effects of encoding strategy and order, but no other effects were found (see Table 3).

##### Reaction time

The analysis of the reaction time data showed main effects of encoding strategy (multimodal < unimodal), time (1,000-ms display time < 1,500-ms display time), order (2nd array test-relevant < 1st array test-relevant), and age (young < older adults). No interaction effects were found (see Table 4).

### Discussion

Experiment 1 showed that aging was indeed associated with declines in working memory performance. Young adults performed significantly better and faster on the present visuospatial working memory task than did older adults. We suggest that age-related declines in working memory capacity can explain this effect of age. More interestingly, for both age groups, a multimodal encoding strategy led to better and faster performance than did a unimodal encoding strategy. Also, both age groups performed better on trials in which the second array was tested than when the first array was tested. However, these main effects seemed to be qualified by an interaction between time, encoding strategy, and order. Follow-up analyses indicated that the positive effect of multimodal encoding was most pronounced in trials with a 1,000-ms display time in which the second array was test relevant, and in trials with a 1,500-ms display time in which the first array was test-relevant (see Fig. 2).

However, multimodal encoding did not compensate for the age-related declines in working memory (i.e., the effects of encoding strategy were similar for young and older adults). We suggest that the present task was more challenging for the older adults, and the task demands might have exceeded their working memory capacity. This could have limited the effect of pointing in this group. Therefore, it is possible that for the older adults, a further offloading of working memory would be needed in order to obtain an optimal effect of pointing.

Although the older adults were slower to point during encoding than were the young adults, the pointing reaction times of the older adults (*M* = 668.14 ms, *SD* = 54.67) showed that they were well able to respond within the 1,000-ms display time. This difference in pointing reaction times during encoding could have been a confounding variable in terms of stimulus exposure. However, older adults did not benefit from the longer stimulus exposure time in terms of performance accuracy; the young adults still outperformed them.

In Experiment 2, we aimed to find out whether offloading working memory by cueing would add to the effect of encoding strategy in the present paradigm, especially for older adults. In addition, this could also provide more insight into whether the claim of Chum et al. (2007), who explained the effect of order as an effect of temporal proximity, holds true, or whether there is some merit to our alternative suggestion that retroactive interference (i.e., memory for the first array being disturbed by the presentation of the second array) also plays a role in the effect of order. If the alternative explanation holds true, then decreasing retroactive interference by cueing, especially by cueing before encoding (because then the second array could simply be ignored, and the “new information” would not access working memory), would improve performance on trials in which retroactive interference would take place (i.e., trials in which the first array was task-relevant). Hence, the effect of order (i.e., working memory performance being superior for the second array relative to the first) would be reduced.

## Experiment 2

In Experiment 2, we investigated whether cueing relevance would add to the effect of multimodal encoding and decrease the effect of order found in Experiment 1.

### Method

#### Participants

Here the participants were 32 young adults (21 women, 11 men; *M*_age_ = 19.8 years, *SD* = 1.5, age range 17–23 years) and 26 older adults (17 women, nine men; *M*_age_ = 65.4 years, *SD* = 3.4, age range 60–71 years). The recruitment procedure and reward for the participants were identical to those aspects of Experiment 1. None of the participants in Experiment 2 had participated in Experiment 1.

#### Materials and procedure

**Fig. 3:**
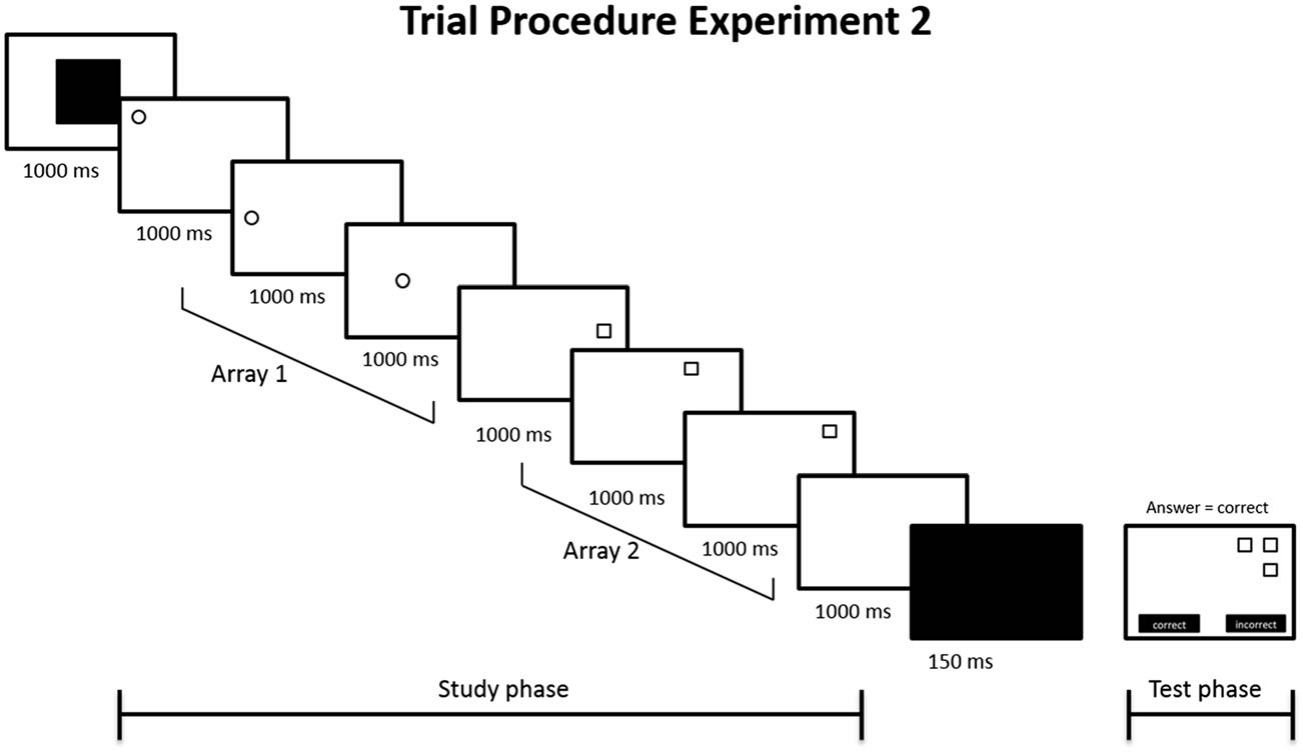
Trial procedure of Experiment 2, depicting an example of a trial in the access condition (cue preceding the encoding phase), with a blank screen presented for 1,000 ms after the encoding phase

For Experiment 2, we used the same materials and procedure as in Experiment 1, except for two changes. First, because Experiment 1 had shown that older adults were well able to make the pointing encoding response within 1,000 ms, only stimulus display times of 1,000 ms were used in Experiment 2. Second, visual cues were presented for 1,000 ms either immediately before or after the encoding phase (see Fig. 3). Depending on the cue condition (before or after encoding), a blank screen was presented for 1,000 ms before or after the encoding phase, to keep the times between the encoding and test phases equal between the cueing conditions (see Fig. 3).

### Results

We analyzed the accuracy and reaction time data with a mixed 2 × 2 × 2 × 2 ANOVA with the within-subjects factors Encoding Strategy (multimodal vs. unimodal), Order (first vs. second array relevant), and Cue Position (before vs. after the encoding phase), and the between-subjects factor Age Group (young vs. older adults). All of the means and standard deviations for accuracy (as percentages) and reaction times (in milliseconds) of Experiment 2 can be found in Table 5. As in Experiment 1, only significant effects are discussed here; statistics for the analyses of Experiment 2 can be found in Tables 6 (accuracy) and 7 (reaction times).

**Table 5:** Means (and *SD*s) for accuracy (Acc) and reaction times (RT) in Experiment 2

			Young Adults (*n* = 32)				Older Adults (*n* = 26)			
			Cue Before		Cue After		Cue Before		Cue After	
	Order		*M*	*SD*	*M*	*SD*	*M*	*SD*	*M*	*SD*
Pointing	1st	Acc (%)	85.07	13.97	77.93	13.24	83.17	14.55	74.04	18.00
	1st	RT (ms)	822	417	1,378	1,008	966	289	1,174	355
	2nd	Acc (%)	90.35	9.33	91.61	12.05	87.02	10.89	86.54	12.21
	2nd	RT (ms)	664	251	639	301	1,017	298	1,024	333
Observing	1st	Acc (%)	80.93	13.43	79.30	16.12	74.04	17.29	73.08	17.21
	1st	RT (ms)	767	305	713	295	1,230	430	1,175	474
	2nd	Acc (%)	91.67	10.90	87.33	13.39	82.21	19.42	76.92	19.27
	2nd	RT (ms)	602	239	658	316	1,124	407	1,213	427

**Table 6:** Statistics of the analyses on performance accuracy in Experiment 2

Analysis	Factor(s)	*df*	*MSE*	*F*	*p*	*η*_p_^2^
Omnibus test	**A**	**1, 56**	**0.05**	**7.88**	**.007**	**.12**
(E × O × C × A)	**E**	**1, 56**	**0.01**	**11.91**	**.001**	**.18**
	**E × A**	**1, 56**		**4.61**	**.036**	**.08**
	**O**	**1, 56**	**0.02**	**38.39**	**<.001**	**.41**
	O × A	1, 56		0.77	.384	.01
	**C**	**1, 56**	**0.02**	**6.56**	**.013**	**.11**
	C × A	1, 56		0.14	.712	<.01
	E × O	1, 56	0.01	0.27	.602	<.01
	E × O × A	1, 56		0.15	.634	<.01
	E × C	1, 56	0.01	0.14	.711	<.01
	E × C × A	1, 56		0.15	.697	<.01
	O × C	1, 56	0.02	0.99	.323	.02
	O × C × A	1, 56		0.02	.892	<.01
	**E × O × C**	**1, 56**	**0.02**	**5.52**	**.022**	**.09**
	E × O × C × A	1, 56		0.03	.855	<.01
Follow-up 1.1: A = Young	E	1, 31	0.01	1.06	.311	.03
Follow-up 1.2: A = Older	**E**	1, **25**	**0.02**	**12.57**	**.002**	**.34**
Follow-up 2.1: E = Multimodal	A	1, 57	<0.01	3.44	.069	.06
**Follow-up 2.2: E = Unimodal**	**A**	1, **57**	**0.01**	**8.99**	**.004**	**.14**
Follow-up 3.1: O = First, C = Before	E	1, 57	0.02	7.05	.010	.11
Follow-up 3.2: O = Second, C = Before	E	1, 57	0.02	0.02	.891	<.01
Follow-up 3.3: O = First, C = After	E	1, 57	0.01	0.52	.475	<.01
**Follow-up 3.4: O = Second, C = After**	**E**	**1, 57**	**0.02**	**8.35**	**.005**	**.13**

A = Age Group (young vs. older adults); E = Encoding Strategy (pointing vs. observation only); O = Order (test stimulus is first vs. second array); C = Cue (before vs. after encoding). Significant effects are printed in boldface

**Table 7:** Statistics of the analyses on reaction times in Experiment 2

Analysis	Factor(s)	*df*	*MSE*	*F*	*p*	*η*_p_^2^
Omnibus test	**A**	**1, 56**	**399,481.85**	**51.35**	**<.001**	**.12**
(E × O × C × A)	**E**	**1, 56**	**84,950.62**	**5.21**	**.026**	**.18**
	**E × A**	**1, 56**		**8.23**	**.006**	**.08**
	**O**	**1, 56**	**80,998.14**	**7.53**	**.008**	**.41**
	O × A	1, 56		1.41	.241	.01
	C	1, 56	110,623.27	0.12	.735	.11
	C × A	1, 56		2.80	.100	.05
	E × O	1, 56	58,723.72	<0.01	.957	<.01
	E × O × A	1, 56		0.09	.766	<.01
	E × C	1, 56	53,473.18	<.01	.958	<.01
	**E × C × A**	**1, 56**		**4.16**	**.046**	**.07**
	O × C	1, 56	75,667.92	0.69	.408	.01
	O × C × A	1, 56		1.96	.167	.03
	E × O × C	1, 56	69,486.06	2.91	.094	.05
	E × O × C × A	1, 56		3.24	.077	.06
Follow-up 1.1: C = 1	E	1, 56	33,166.53	3.46	.068	.06
	**A**	1, 56	**125,463.29**	**31.45**	**<.001**	**.36**
	**E × A**	**1, 56**		**12.89**	**.001**	**.19**
Follow-up 1.2: C = 2	E	1, 56	36,045.37	2.96	.091	.05
	**A**	**1, 56**	**129,589.27**	**49.89**	**<.001**	**.47**
	E × A	1, 56		0.92	.341	.02
Follow-up 2.1: C = 1; A = Young	E	1, 31	31,984.23	1.73	.198	.05
Follow-up 2.2: C = 1; A = Older	**E**	**1, 25**	**34,632.58**	**12.88**	**.001**	**.34**

A = Age Group (young vs. older adults); E = Encoding Strategy (pointing vs. observation only); O = Order (test stimulus is first vs. second array); C = Cue (before vs. after encoding). Significant effects are printed in boldface

#### Experimental task

##### Encoding

The older participants (*M* = 638.14 ms, *SD* = 36.57) were slower to point at the figures than were the young adults (*M* = 557.06 ms, *SD* = 34.51), *F*(1, 75) = 77.52, *MSE* = 1,260.21.14, *p* < .001, *η*_p_^2^ = .58.

##### Test

Analysis of the accuracy data revealed main effects of encoding strategy (multimodal > unimodal), order (2nd array tested > 1st), cue (before > after), and age group (young > older adults). Interactions were also found between encoding strategy and age group and between encoding strategy, order, and cue. No other interactions were statistically significant (see Table 6, Omnibus test).

The interaction between encoding strategy and age group was further explored with four repeated measures ANOVAs: one for each age group separately, with Encoding Strategy as a within-subjects factor, and one for each encoding strategy separately, with Age Group as a between-subjects factor. The analysis of the young adults’ performance data showed no effect of encoding strategy; that is, for young adults, pointing no longer had a beneficial effect as compared with observation only (see Table 6, Follow-up 1.1). In contrast, the analysis of the older adults’ performance data did show an effect of encoding strategy; older adults were more accurate in the multimodal than in the unimodal encoding condition (see Table 6, Follow-up 1.2). The analysis of the young and older adults’ performance accuracy in the multimodal encoding condition revealed that the older adults’ performance was equal to that of the young adults (see Table 6, Follow-up 2.1), whereas in the unimodal encoding condition, the older adults’ performance was lower than that of the young adults (see Table 6, Follow-up 2.2).

**Fig. 4:**
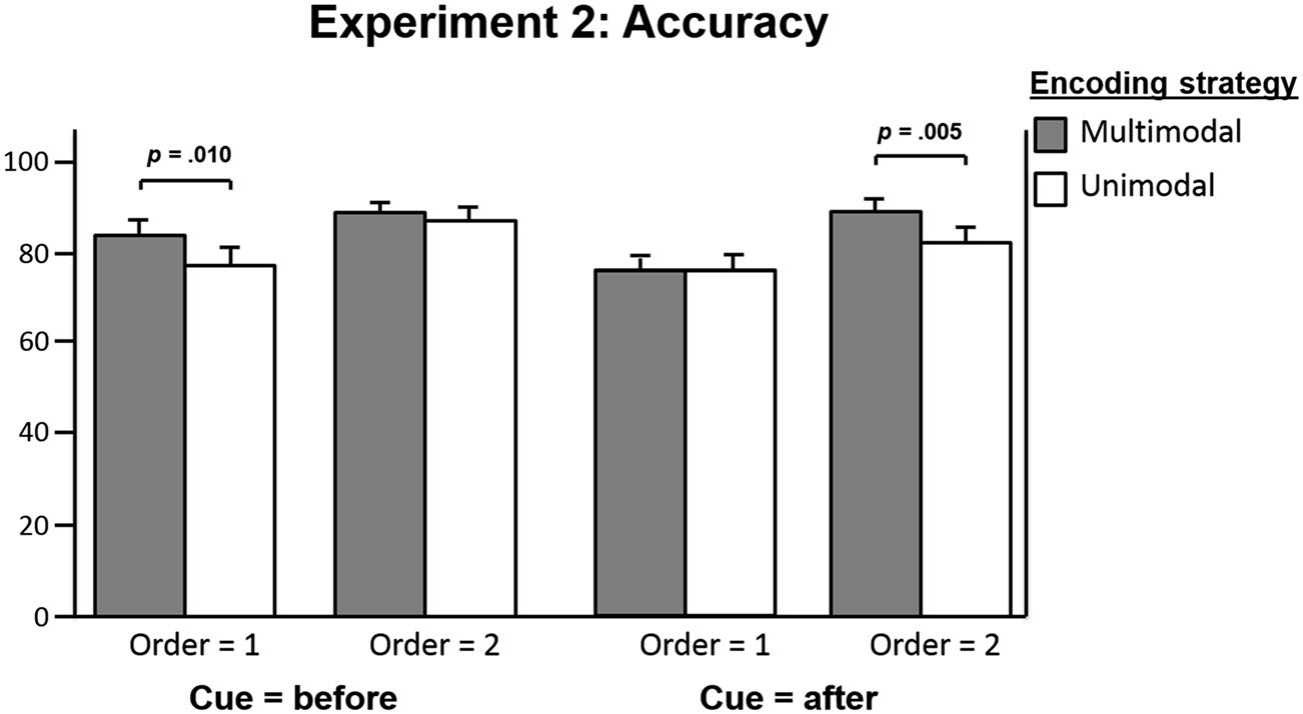
Experiment 2: Interaction between encoding strategy, order, and cue

The interaction of encoding strategy, order, and cue was further analyzed with four repeated measures ANOVAs, testing the effect of encoding strategy (1) in the access condition when the first array was tested, (2) in the access condition when the second array was tested, (3) in the deletion condition when the first array was tested, and (4) in the deletion condition when the second array was tested (see Table 6, Follow-ups 3.1–3.4, and Fig. 4). The results showed an effect of encoding strategy in the deletion condition when the second array was tested (multimodal, *M* = 89.33 %, *SD* = 12.28; unimodal, *M* = 82.67 %, *SD* = 16.96), but not when the first array was tested (multimodal, *M* = 76.18 %, *SD* = 15.53; unimodal, *M* = 76.51 %, *SD* = 16.76). In the access condition, a marginally significant effect (*p* = .010, Bonferroni-corrected significance level of .006) of encoding strategy was found when the first array was tested (multimodal, *M* = 84.22 %, *SD* = 14.14; unimodal, *M* = 77.84 %, *SD* = 15.53), but not when the second array was tested (multimodal, *M* = 88.86 %, *SD* = 10.10; unimodal, *M* = 87.43 %, *SD* = 15.89).

However, with these results we were not able to disentangle the effect of retroactive interference from the effect of encoding strategy. It is possible that pointing and cueing also might have interfered with each other (e.g., pointing to one array and cueing the other, as compared with cueing and pointing to the same array). To check this, six comparisons with an adjusted alpha level of .05/6 = .008 for all possible pairs were conducted, between cueing and pointing to the first array (C1P1, *M* = 78.98 %, *SD* = 11.28), cueing and pointing to the second array (C2P2, *M* = 88.34 %, *SD* = 9.83), cueing the first array but pointing to the second (C1P2, *M* = 75.75 %, *SD* = 12.70), and cueing the second array but pointing to the first (C2P1, *M* = 86.00 %, *SD* = 12.78).

The results showed that performance accuracy was (1) lower on C1P1 than on C2P2, *t*(57) = –5.69, *p* < .001, *d* = *–*1.16; (2) lower on C1P1 than on C2P1, *t*(57) = –4.13, *p* < .001, *d* = *–*0.58; (3) lower on C1P2 than on C2P2 than on C1P2, t(57) = -7.10, p < .001, d = -1.39; and (4) lower on C1P2 than on C2P1, *t*(57) = –5.78, *p* < .001, *d* = –0.80. No performance differences were found between (5) C1P1 (*M* = 78.98, *SD* = 11.28) and C1P2 (*M* = 75.75, *SD* = 12.70), *t*(57) = 1.94, *p* = .057, *d* = 0.26, or between (6) C2P2 (*M* = 88.34, *SD* = 9.83) and C2P1 (*M* = 86.00, *SD* = 12.78), *t*(57) = 1.56, *p* = .124, *d* = 0.27. These results show that performance was higher when the second array was cued than when the first array was cued, irrespective of which array had been pointed at. This suggests that pointing did not interfere with cueing (i.e., whether or not the array cued was also pointed at). Results are all rephrased per Ms given above. Please check. Note that final t value is also made negative as per means.Thank you for this correction. This is correct. I do however, have a question about the changes made in the presentation of the means behind some of the analyses, but not all. In my version I chose to present the means once in the paragraph above and not in the result section in this paragraph. Now I see that the means are given in the description of the null effects, but not in the description of the difference effects. This seems a bit inconsistent to me. However, I do realise that being the first author and having read this manuscript so many times, might have clouded my judgment on what is best from a reader's perspective. So please take this comment as a notification, to see what you find best.

The analysis of the reaction time data showed main effects of encoding strategy, order, and age, but not of cue. Significant interaction effects were found for encoding strategy and age group, and for encoding strategy, cue, and age group. No other interaction effects were significant (see Table 7, Omnibus test).

The interaction of encoding strategy, cue, and age group was further explored by conducting repeated measures ANOVAs for each cueing condition separately, with Encoding Strategy as a within-subjects factor and Age Group as a between-subjects factor. Analysis of the trials in the access condition revealed no effect of encoding strategy, an effect of age group (young < older), and an interaction between encoding strategy and age group (see Table 7, Follow-up 1.1). We further explored this interaction between encoding strategy and age group by conducting a repeated measures ANOVA for each age group separately, with Encoding Strategy as a within-subjects factor. These analyses revealed an effect of encoding strategy in older adults (see Table 7, Follow-up 2.1), but not in young adults (see Table 7, Follow-up 2.2). These results reflect that in the access condition, the older but not the young adults were faster to recognize the multimodally than the unimodally encoded arrays.

Analysis of the trials in the deletion condition revealed no effect of encoding strategy, an effect of age group (young < older), but no interaction between encoding strategy and age group (see Table 7, Follow-up 1.2). These results showed that on trials with cues presented after encoding, young adults were faster to respond than older adults.

### Discussion

In Experiment 2, the effect of encoding strategy was no longer present in young adults, presumably because they adopted a different learning strategy than in Experiment 1 in response to the cues provided. For older adults, however, pointing still had a beneficial effect. In fact, they performed equally as well as the young adults in the multimodal encoding condition, but more poorly in the unimodal encoding condition. We will elaborate more on this finding in the General Discussion.

For both age groups, in the deletion condition, an effect of encoding (multimodal > unimodal) only emerged when the second array was tested, which is in line with the interaction found in Experiment 1, which also showed an effect of encoding strategy (multimodal > unimodal) when the second array was task-relevant. In the access condition, however, a trend was found toward an effect of encoding strategy when the first array was tested. Although this effect was only marginally significant after the Bonferroni correction, it does provide further insight into the nature of the significant interaction between encoding strategy, order, and cue. It seems that in combination with cueing before encoding, pointing possibly ameliorated the negative effect of temporal decay, which suggests that retroactive interference also might play a role in the effect of order. In addition, for both age groups, performance differed depending on which array was cued (i.e., performance was better on the second than on the first cued array), regardless of which array had been pointed at. No performance differences were found between conditions that only differed in which array had to be pointed at. This suggests that pointing did not interfere with cueing.

## General discussion

In the present study, we aimed to replicate the findings of Chum et al. (2007) that pointing facilitates visuospatial working memory in young adults, and to investigate whether any positive effects would also apply to older adults (Exp. 1). Second, we investigated whether cueing would add to the effect of encoding strategy and influence the effect of order (i.e., the time lag between the encoding and test phases) on performance (Exp. 2) and retroactive interference.

In line with the hypotheses, in Experiment 1 we replicated the findings of Chum et al. (2007), which revealed that a multimodal as compared with a unimodal encoding strategy led to better visuospatial working memory performance in young than in older adults. Consistent with previous evidence showing age-related declines in working memory functioning (e.g., Salthouse & Babcock, 1991), Experiment 1 showed that young adults outperformed older adults in general.

For the positive effect of pointing on visuospatial working memory in the young and older adults in Experiment 1, we adopt the explanation of Chum et al. (2007), who used the selection-for-action hypothesis of Allport (1989) that pointing aids selective attentional processes during encoding. Selective attention has been associated with working memory and is even said to influence working memory performance (Gazzaley & Nobre, 2012). Cognitive control has been proposed to underlie selective attention and working memory performance (Gazzaley & Nobre, 2012) and was found to decline with aging (Egner & Hirsch, 2005). Cognitive control can be seen as an internal control system managed by the brain (i.e., prefrontal areas) that signals and amplifies task relevance, by modulating the neural activity in sensory areas depending on the relevance of a stimulus (Egner & Hirsch, 2005). We suggest that in our study pointing toward the stimulus locations could enhance working memory and selective attention, because it served as an external control system, guiding attention.

We suggest that pointing is a very suitable way to enhance older adults’ working memory performance, because it is rather effortless. This idea comes from Geary (2008, 2012), who stated that there are two kinds of knowledge, named biologically primary and secondary knowledge. *Primary* knowledge consists of information that humans have evolved to process and understand automatically, including action and action understanding (and imitation; Paas & Sweller, 2012). In contrast, *secondary* knowledge is only gained by explicit learning, which demands effort and conscious cognitive processing. We suggest that, when indexing and encoding spatial information, pointing toward locations is based on primary knowledge. This claim is supported by the fact that pointing gestures are among the most robust human gestures, and that young children point toward objects and locations even before they are able to speak (Iverson & Goldin-Meadow, 2005). Because pointing is a motoric and body-based action, this would be a rather effortless (requiring little to no working memory capacity) manner to add an extra memory code through which retrieval can occur.

Although the results found by Chum et al. (2007) were replicated in Experiment 1, the multimodal encoding strategy was not found to compensate for the age-related declines in working memory, because the effects of encoding strategy were similar for young and older adults, and the young adults outperformed the older adults in general. Important to mention is that keeping all figures in working memory until the test phase and then selectively suppressing the irrelevant figures (deletion function) was probably more challenging for older than for young adults, because of age-related declines in working memory (e.g., Cansino et al., 2011; Salthouse & Babcock, 1991) and interference control (e.g., Cansino et al., 2011; Houx et al., 1993; Stoltzfus et al., 1993).

In Experiment 2, we investigated whether offloading working memory by cueing in the present paradigm would add to the effect of encoding strategy, especially in older adults, and ameliorate the effect of order. The first main finding of Experiment 2 was an interaction between age group and encoding strategy, in that a multimodal encoding strategy improved older adults’ performance, bringing it up to the level of young adults. The performance of young adults, however, did not differ between encoding strategies. Although the interaction between age group and encoding strategy did not interact with cueing, the only difference between Experiments 1 and 2 was the addition of the cues. This suggests that cueing might have had some influence on the compensatory effect of multimodal encoding on older adults’ visuospatial working memory performance. However, because we found no interaction between age group and cueing, this result is not in line with the findings of Cansino et al. (2011), who showed that young adults benefit from cueing both before (access) and after (deletion/suppression) encoding, and older adults only from cueing before (access), suggesting that older adults have specific problems with the deletion/suppression of irrelevant information in working memory. A possible explanation can be found in research showing that low working memory span is related to poor source monitoring (e.g., Lilienthal, Rose, Tamez, Myerson, & Hale, 2015). For example, Lilienthal et al. (2015) found that individuals with low working memory spans have difficulty with distinguishing between relevant and irrelevant information, rather than with suppressing irrelevant information, at retrieval. Because of age-related declines in working memory functioning (Salthouse & Babcock, 1991), older adults have a smaller working memory span than young adults. Therefore, it is possible that cueing in addition to pointing was especially beneficial for older adults’ working memory performance. The improved source monitoring might have prevented working memory overload (i.e., cognitive overload; see Paas et al., 2003) in the older adults, and thereby added to the effects of pointing on working memory performance in both cueing conditions.

A possible explanation for why young adults did not benefit from multimodal encoding when cues were added in Experiment 2 is that the cues probably made the pointing redundant in this group. Although Chum et al. (2007) found that the effect of pointing was strongest for their smallest arrays, these arrays still contained six figures, which is challenging for working memory (Cowan, 2010). However, we suggest that the cueing reduced the working memory load to a level that was not challenging anymore for the young adults’ working memory, and therefore the effect of pointing disappeared in this group.

The second main finding in Experiment 2 was an interaction between encoding strategy, order, and cue. This finding reflects that in the deletion condition, pointing had a beneficial effect only when the second array was tested (which is in line with the interaction between encoding strategy and order found in Exp. 1). In the access condition, however, the effect of encoding was nearly significant for performance when the first array was tested. Although we need to be cautious with interpreting this finding, since it was only marginally significant after the (conservative) Bonferroni correction, it does suggest that not only temporal proximity, but also retroactive interference, may have been responsible for the effect of order found by Chum et al. (2007) and in the present Experiment 1.

The positive effects of a multimodal encoding strategy on visuospatial working memory for young and older adults that we found in Experiment 1 suggest that the simple act of pointing during the encoding of stimulus locations can improve working memory performance in both young and older adults. However, the most important result was that if the relevant stimuli were cued, additionally pointing to them seemed to compensate for age-related declines in working memory performance (Exp. 2). The present finding that the working memory performance of older adults can benefit from contextual cues is contradictory to some important aging studies that have shown age-related declines in context processing (Braver & Barch, 2002; Braver, Satpute, Rush, Racine, & Barch, 2005). Instead of enhancing inhibitory processes (suppressing irrelevant stimuli), Braver and Barch (2002) proposed that contextual cueing enhances selective processes (biasing attention toward relevant stimuli). In fact, some researchers have even proposed an account of interference effects that does not include inhibition (MacLeod, Dodd, Sheard, Wilson, & Bibi, 2003). Braver et al. (2005) showed that healthy aging is related to a decline in context representation and updating. More specifically, using a continuous-performance task (CPT), Braver et al. showed that older adults were outperformed by young adults on trials in which they had to respond to a target that was preceded by an invalid cue, but they outperformed young adults on trials in which a valid cue preceded a nontarget. These findings were interpreted as a decreased sensitivity for contextual cues, and was taken as evidence that aging is related to a decline in context processing. Important to note is that the CPT (pseudo)randomly presented valid and invalid cues, and these cues were (pseudo)randomly followed by targets or nontargets. Contextual representation and updating in such a task imposes much more load on working memory than did the present task. The task that we used had a clear trial structure that included only cues that validly predicted which figures’ locations would be tested. Although we acknowledge the existence of age-related declines in context representation and updating, we suggest that these age-related declines would be more apparent in the CPT because this task puts more load on working memory (in terms of predictability and context updating) than did the task used in Experiment 2. However, it is clear that further experimentation will be needed for us to find out whether the effects of cueing in combination with pointing on older adults’ working memory can best be explained by the inhibition of nontarget information or the enhancement of target information.

A limitation of the present study is that, from the results, we cannot disentangle the individual effects of pointing and cueing. However, the present study focused on replicating the effect of pointing on young adults’ visuospatial working memory and finding out whether a similar effect would be present in older adults (Exp. 1). Furthermore, we investigated whether the claim made by Chum et al. (2007), that the effect of order in the present paradigm was caused by the temporal delay (Exp. 2), was true, or whether the interference of the irrelevant subset entering working memory also influenced performance. Therefore, we added cues in the present paradigm. Nevertheless, it would be an interesting idea for future research to investigate the effects of pointing and cueing on visuospatial working memory separately. In addition, it would also be interesting to purely vary the temporal distance (without presenting interfering stimuli) between encoding and test in a similar paradigm, to find out whether the effect of cueing would still be present.

In conclusion, the present study showed that the visuospatial working memory performance of both young and older adults improved using a multimodal as compared with a unimodal encoding strategy. However, the most important finding was that a multimodal encoding strategy only compensated for age-related declines in working memory performance when the relevant stimuli were visually cued. This last finding seems to suggest that working memory load, rather than just temporal proximity, is what was responsible for the effect of order found in both Chum et al. (2007) and our Experiment 1. These findings are especially interesting from a cognitive-aging perspective, because they suggest that (at least in the present paradigm) gestures and visual cues can be used as tools to compensate for age-related declines in visuospatial working memory performance.
